# Identification of Subgingival Periodontal Pathogens and Association with the Severity of Periodontitis in Patients with Chronic Kidney Diseases: A Cross-Sectional Study

**DOI:** 10.1155/2015/370314

**Published:** 2015-04-02

**Authors:** Fidan Bahtiar Ismail, Gener Ismail, Anca Silvia Dumitriu, Catalin Baston, Vlad Berbecar, Roxana Jurubita, Andreea Andronesi, Horia Traian Dumitriu, Ioanel Sinescu

**Affiliations:** ^1^Department of Periodontology, “Carol Davila” University of Medicine and Pharmacy, Dionisie Lupu Street No. 37, District 1, 020021 Bucharest, Romania; ^2^Center of Internal Medicine-Nephrology, Fundeni Clinical Institute, 258 Fundeni Street, District 2, 022328 Bucharest, Romania; ^3^Center for Uronephrology and Renal Transplantation, Fundeni Clinical Institute, 258 Fundeni Street, District 2, 022328 Bucharest, Romania

## Abstract

*Background*. The aim of our study was to assess the subgingival profile of 9 periodontal pathogens, by means of real-time PCR, in a group of predialysis chronic kidney disease patients with and without periodontal disease and to identify the risk factors associated with periodontal disease in these patients. *Material and Methods*. This is a single centre cross-sectional cohort study performed on 70 CKD patients. Patients received a full-mouth periodontal examination and the following parameters were assessed: periodontal pocket depth (PPD), clinical attachment level, bleeding on probing, and plaque index; subgingival biofilm samples were collected from the deepest periodontal pocket of each quadrant and were pooled in one transporting unit. Clinical data were drawn from the medical file of the patients. *Results*. *T. denticola* (*P* = 0.001), *T. forsythia* (*P* < 0.001), and *P. micros* (*P* = 0.003) are significantly associated with periodontal disease in CKD subjects but in a multivariate model only age and *T. forsythia* remain independent risk factors for periodontal disease in patients with CKD. *Conclusions*. In our cohort, age and *T. forsythia* are independently associated with periodontitis in CKD patients. Within the limits of this study, CKD was not significantly associated with a particular subgingival periodontal pathogens profile in periodontitis patients.

## 1. Introduction

Chronic kidney diseases (CKD) are a global public health issue with increasing prevalence, incidence, and adverse outcomes. Prevalence of CKD evaluated in the NHANES IV cohort [1] was surprisingly elevated: 13%; similar results were reported also in other geographical areas such as Australia (16%) [2] and China (13%) [3]. Periodontal diseases comprise a group of inflammatory diseases which affect the supporting tissues of the teeth in individuals who present a disease susceptible background. Recently, numerous studies [4–7] on the association between periodontal diseases and CKD have emerged and have attempted to demonstrate that there is a bidirectional relationship between periodontal disease and CKD. There are some mechanisms considered to be involved in the bidirectional association between periodontal disease and CKD: the capability of the proinflammatory cytokines to induce endothelial dysfunction and atherogenesis [8], molecular mimicry of bacterial heat shock proteins such as GroEl 60 with human heat shock proteins secreted in response to endothelial injury which could induce atheroma formation [9], and increased levels of serum antibody to periodontal pathogens which reflect systemic dissemination of these organisms and “vascular and hepatic activation” [10].

Nowadays, the microbial etiology of periodontal diseases is unquestionable because of the key role of dental biofilm in initiation of inflammation of periodontal tissues but the presence of microorganisms is not sufficient for the disease to take place; the host response, modulated by a variety of local, systemic, and environmental conditions and modifiable or unmodifiable factors, is the one which decides whether the inflammation following plaque accumulation manifests and remains as gingivitis or progresses to periodontitis.

There are only a few studies performed on subgingival microbial flora in CKD patients; therefore, the aim of our study was to assess the subgingival microbial profile of predialysis chronic kidney disease patients with and without periodontal disease and to identify the risk factors associated with periodontal disease in patients with CKD.

## 2. Material and Methods

### 2.1. Study Design

This is a single centre cross-sectional study using prospectively collected data. Recruitment of the study population was performed during January 2014 and May 2014. This cohort study included consecutive patients aged >18 years with chronic kidney disease stages 3, 4, and 5 (not on haemodialysis or peritoneal dialysis) recruited from the Department of Internal Medicine-Nephrology, Fundeni Clinical Institute. The exclusion criteria were as follows: less than 15 teeth, anticoagulation therapy, acute infections or history of antibiotic treatment in the past 3–6 months, use of corticosteroids and/or other immunosuppressive agents, patient who had undergone initial or surgical periodontal therapy in the last 6 to 12 months, patients on dialysis, and viral infections (HIV, HBV, and HCV).

After a preliminary oral examination of 122 patients, 27 patients did not fulfill inclusion criteria, 10 patients did not sign the informed consent, and 15 patients refused comprehensive periodontal examination.

The clinical characteristics included age, sex, body mass index, systolic and diastolic blood pressure, serum total cholesterol level, serum creatinine level, glomerular filtration rate, haemoglobin level, and serum albumin level.

GFR was estimated from serum creatinine, using the four variables MDRD (modification of diet in renal disease) study equation [11].

Smoking habits were recorded only as smoker or nonsmoker, with no regard to the number of cigarettes or to the status of former smoker.

Patients received a full-mouth periodontal examination at six sites on every tooth with a calibrated periodontal probe (UNC 15). We have recorded the following periodontal parameters: number of missing teeth (with the exception of third molars), O'Leary plaque scores, presence or absence of bleeding on probing (BoP), periodontal pocket depth (PPD), and clinical attachment level (CAL) measured and rounded to the nearest highest millimetre.

Chronic periodontitis was defined using CDC/AAP (Centers for Disease Control and Prevention/American Academy of Periodontology) definition according to Page and Eke [12] for moderate and severe periodontitis. According to this, moderate periodontitis was considered as at least two interproximal sites with clinical attachment loss equal to or more than 4 mm (not on the same tooth) or at least two interproximal sites with periodontal probing depth equal to or greater than 5 mm (not on the same tooth); severe periodontitis was defined as at least two interproximal sites with CAL ≥6 mm and one or more interproximal sites with PD ≥5 mm (not on the same tooth). Third molars were excluded.

The study protocol was approved by the local ethics committee.

### 2.2. Collection of Subgingival Biofilm

Collection of subgingival biofilm was performed by means of 4 sterile paper points (one for each quadrant) which were inserted in the deepest, bleeding on probing periodontal pocket of the quadrant, after isolation, drying, and removal of supragingival biofilm. The paper points were maintained for 20 seconds and after that they were transferred in one transportation tube (pool sample), according to the manufacturer's protocol. The tubes were then enveloped and sent to the laboratory for polymerase chain reaction (PCR) analysis.

Microbial analysis was performed for 9 periodontal pathogens (*Aggregatibacter actinomycetemcomitans* (Aa),* Porphyromonas gingivalis* (Pg),* Treponema denticola* (Td),* Tannerella forsythia* (Tf),* Prevotella intermedia* (Pi),* Peptostreptococcus micros* (Pm),* Fusobacterium nucleatum* (Fn),* Eubacterium nodatum* (En), and* Capnocytophaga gingivalis* (Cg)). We have used a commercially available real time PCR kit (LightCycler 480, Roche) which not only identifies the presence of a particular microorganism, but also detects the relative number of that microorganism in the examined probe.

### 2.3. Statistical Analyses

Continuous variables are presented as mean or median (95% confidence intervals), according to their distribution, and categorical variables as percentages. Group comparisons were performed with Student's *t*-test, *χ* ^2^ test, and Mann-Whitney *U* test, as appropriate.

Multivariable-adjusted binomial logistic regression was used to investigate the association between the periodontal disease and the investigated risk factors.

A *P* ≤ 0.05 was considered statistically significant. Analyse-it (Analyse-it Software, Ltd., Leeds, UK) and SPSS version 14 (SPSS Inc., Chicago, IL, USA) software were used to analyse the data.

## 3. Results

The demographic, clinical, and periodontal parameters are presented in Tables 1 and 2.

The study group consisted of 70 predialysis CKD patients with a mean age of 46.3 ± 16.1 years (18–80), 37 (52.9%) of which were males. Serum creatinine levels were 2.6 ± 1.2 mg/dL, serum albumin was 3.6 ± 0.5 g/dL, and eGFR was 28.6 ± 10.8 mL/min/1.73 m^2^ (8–50). The number of patients with diabetes represented only 10% (7 patients) and smokers were 21.4% (15 patients).

In the study group, mean CAL was 3.6 ± 1.1 mm, mean PD was 2.6 ± 0.4 mm, and O'Leary plaque index was 90.6 ± 9%.

The distribution of patients according to the severity of periodontal disease was as follows: 34 (48.5%) patients had severe periodontitis and 12 patients (17.1%) had moderate periodontitis, whereas 20 (28.5%) had gingivitis or mild periodontitis and 4 (5.9%) were free of periodontal disease.

Table 3 shows clinical characteristics of the periodontitis group (moderate and severe forms of periodontal disease) versus periodontitis-free group. There was a statistical significant difference between the 2 groups regarding the mean age of the patients (49.2 ± 16.6 versus 40.8 ± 13.8, *P* = 0.02) and mean eGFR values (27 ± 10.4 mL/min/1.73 m^2^ versus 31.8 ± 11 mL/min/1.73 m^2^, *P* = 0.04).

Frequency of detection and univariate analyses for the association with periodontal disease are shown in Table 4. In our group,* C. gingivalis* was the most prevalent microorganism, with a 97.1% (68 patients) detection frequency, followed by* P. micros* (91.4%, 64 patients),* F. nucleatum* (88.6%, 62 patients),* T. denticola* (85.7%, 60 patients),* P. intermedia* and* T. forsythia* (with 80% and 77.1%, resp.),* P. gingivalis* (72.9%, 51 patients),* E. nodatum* (32.9%, 23 patients), and* A. actinomycetemcomitans* with a 5.9% frequency of detection.* A. actinomycetemcomitans* was identified in only 4 cases and in very small quantities (2.3 × 10^1^ copies); 2 of the patients who were positive for this pathogen had moderate periodontitis while the other 2 had a healthy periodontal tissue.

Taking into consideration the relative number of each bacteria in the whole sample, we have divided our results according to 3 ranges of bacteria quantification (modified after Haffajee [13]) as follows: 0 (undetectable) and ≤10^5^ and >10^5^ copies.  Table 5 describes the distribution of periodontal pathogens according to the 3 levels described above.

The relative number of periodontal pathogens did vary significantly between analysed specimens,* P. intermedia* having the largest variation, between undetectable and 2.5 × 10^6^ copies. Number of copies of* P. gingivalis* varied between undetectable in 19 patients, values under 10^5^ in 22 patients, and values greater than 10^5^ in 29 patients, with the highest number being 9.5 × 10^5^ copies.

In univariate analysis, as compared to those without periodontal disease, the patients with moderate and severe forms of periodontitis were older (mean age 49.2 ± 16.6 versus 40.8 ± 13.8, *P* = 0.02) and had significantly lower values of eGFR (27 ± 10.4 versus 31.8 ± 11, *P* = 0.04) (Table 3). Moreover,* T. denticola* (*P* = 0.001),* T. forsythia* (*P* < 0.001), and* P. micros* (*P* = 0.003) were significantly associated with periodontal disease in CKD subjects, but in multivariate analysis, only age and* T. forsythia* remained independent risk factors for periodontal disease in these patients (Table 6).

## 4. Discussions

Analyses of subgingival biofilm species collected from periodontal patients through cultivation methods have shown that Gram negative microbial species are predominant, 85% being anaerobe or facultative anaerobe, where active lesions were dominated by “*B. intermedius*,* F. nucleatum*,* B. gingivalis*, and* B. forsythus*” [14]. Molecular methods used for detection and identification of subgingival microbiota have also reported that, in periodontal patients, the deepest portion of the periodontal pocket is colonized by motile and Gram − species whereas superficial portions are colonized by Gram + cocci [15].

Subgingival biofilms might work as large reservoirs from which bacteria, bacterial derivatives such as LPS (lipopolysaccharide), heat shock proteins (like GroEl 60), and proinflammatory cytokines permanently flood the bloodstream affecting distant sites and organs [16–20]. Moreover, patients with active and/or aggressive forms of periodontal disease might present a hyperresponsive immune system compared to those with chronic or inactive forms of disease. During its active progression, the periodontal lesion could trigger a complex immune and inflammatory challenge which explains the higher serum levels of IgG antibody against* P. gingivalis* that are documented in patients diagnosed with periodontitis [21]. Studies have documented both the local and systemic inflammatory responses by analysing the cytokine profile in gingival crevicular fluid, in saliva, and in serum; increased serum levels of CRP in periodontitis patients were reported and decrease of its concentrations after initial periodontal therapy [22–26].

Consequently, when evaluating the possible role played by periodontal diseases in systemic inflammation we should also have to take into account the severity of disease and discriminate between active and inactive forms, not only between periodontally healthy and diseased subjects.

At the same time, CKD is characterized by a persistent systemic inflammatory status even in absence of a particular cause, so it is plausible to assume that the inflammatory response induced by periodontal disease could add to the total inflammatory burden in these patients.

Fisher and colleagues [27] have stated that there is a bidirectional relationship between periodontal disease and CKD, an association mediated by diabetic status and hypertension. On the other hand, in the search for explaining the periodontal systemic relationship, Seymour et al. [28] have also discussed the hypothesis of common susceptibility which comprises a genetically based phenotype leading to a higher risk of both infection and atherogenesis and would also involve a hyperinflammatory phenotype [5].

An ARIC (atherosclerosis risk in communities) derived study [29] has evaluated the association between periodontal disease and CKD and was the “first study to show an association of periodontal disease with prevalent renal insufficiency” defined as eGFR less than 60 mL/min/1.73 m^2^ with an odds ratio of 2.00 (95% confidence interval, 1.23 to 3.24). The dental ARIC study of the same group [9] has evaluated serum antibodies (immunoglobulin-*γ* IgG) to 8 periodontal pathogens and has shown that CKD with eGFR <60 mL/min/1.73 m^2^ is significantly associated with high levels of serum IgG to* P. gingivalis*,* T. denticola,* and* A. actinomycetemcomitans* with an odds ratio of 1.6 to 1.8 (*P* < 0.05).

Kshirsagar also speculates on the possibility that periodontal pathogens might also produce direct damage to the renal cells or to renal vessels. Because of the persistent low-level bacteraemia that characterizes the patients with periodontitis, bacteria reach the glomerulus where they are filtered out and also might invade the endothelium or mesangial cells and matrix [9]. Further studies are needed to demonstrate bacterial invasion of the kidney tissues, although* P. gingivalis* has been shown to invade aortic and coronary endothelial cells [30, 31].

Studies by Buhlin et al. [32] and Grubbs et al. [33] have suggested that CKD patients have a reduced concern in oral hygiene and that addressability to oral healthcare providers is low because of the psychological and emotional burden represented by the disease in itself and the long renal replacement therapy sessions.

In our study group, the most prevalent microorganism was* C. gingivalis*, with a 97.1% detection frequency, followed by* P. micros* (91.4%),* F. nucleatum* (88.6%),* T. denticola* (85.7%),* P. intermedia* and* T. forsythia* (with 80% and 77.1%, resp.),* P. gingivalis* (72.9%),* E. nodatum* (32.9%), and* A. actinomycetemcomitans* with a 5.9% frequency of detection. This could be explained by the fact that in CKD patients the uremic milieu creates an alkaline environment which favours multiplication of proteolytic periodontal pathogens. Moreover, patients with CKD might have an increased risk for infections because of the immunocompromised state seen in a uremic milieu [34].

Our method of biofilm collection (by paper point) might also explain the higher detection frequency of red and orange complex pathogens. Studies of Kigure et al. [35] and Noiri et al. [36–39] cited by Socransky and Haffajee [15] have shown that red complex microorganisms are frequently located near the periodontal pocket epithelium while orange complex pathogens are more frequently detected in the free, unattached subgingival plaque.

In our study, we have detected red complex bacteria not only in periodontitis subjects but also in gingivitis subjects (PPD < 3 mm) but in periodontally healthy patients, the only detected pathogens were* C. gingivalis* in 100 (24 patients) and* F. nucleatum* in 50% of the cases. Furthermore, these pathogens had a higher prevalence in the entire study group.* F. nucleatum* plays an important role in the biofilm ecology because of its coaggregation properties with many bacterial species, allowing physical interactions between Gram-positive and Gram-negative pathogens [40]. This pathogen was also implicated in nonoral infections like pleural-pulmonary infections, urinary tract infections, endocarditis, and preterm-birth [41, 42]. It was also identified rather in active sites than in inactive pockets [14].

In our study, univariate analysis showed that* T. denticola* (*P* = 0.001),* T. forsythia* (*P* < 0.001), and* P. micros* (*P* = 0.003) were significantly associated with periodontal disease in CKD subjects. After including these parameters in a multivariable analysis, age and* T. forsythia* remained independent risk factors for periodontal disease in patients with CKD.

Takeuchi et al. [43] have analysed 6 periodontal pathogens and have shown that* P. gingivalis*,* T. denticola,* and* P. nigrescens* were detected more frequently in renal patients than in healthy controls; they suggested that this is due to an inferior oral hygiene level of the renal patients and also due to the relatively higher percentage of diabetes in their renal patients cohort. Similar results were obtained also by Bastos et al. [44] who compared the subgingival microbial profile of healthy and CKD patients (predialytic and dialysis patients), but only* P. gingivalis* and* T. denticola* have reached statistical significance and were associated with greater CAL.

Castillo et al. [45] have evaluated periodontal status and oral microbiological profile of haemodialysis patients but have not found a particular pattern of microbial colonization in these patients; however,* T. forsythia* was the most frequently detected pathogen, followed by* P. nigrescens*,* P. gingivalis*,* P. intermedia,* and* A. actinomycetemcomitans*, but only* P. nigrescens* was significantly correlated with CAL. Still, haemodialysis patients presented greater numbers of periodontal pathogens compared to controls.

Study of Torres et al. [46] has compared the periodontal status and results of BANA test (associated with the three red complex species) in haemodialysis patients and healthy controls with periodontitis; the authors reported that in ESRD patients in haemodialysis, the frequency of positive BANA tests is greater than in controls, even in shallow pockets, suggesting that alkaline uremic milieu favours multiplication of these pathogens. It is noteworthy to evoke the study of Naugle et al. [47] on mixed anaerobic cultures which demonstrated that a rise of the environmental pH from 7 to 7.5 induces a marked proliferation of* P. gingivalis*, from numbers less than 1% of the entire microbial community to reaching predominance of that culture.

There are some important limitations of our study. First, we have compared the periodontal pathogens profile within a CKD group and not with healthy controls; in addition, the number of patients enrolled in the study was limited because of financial restrictions and finally, the cross-sectional analysis prevented us from testing a directional relationship between CKD and periodontitis.

## 5. Conclusions

Our data has demonstrated that* T. denticola*,* T. forsythia,* and* P. micros* are frequently detected in CKD patients with both moderate and severe forms of periodontitis. Also, age and* T. forsythia* are independent risk factors for periodontitis in CKD patients. However, the periodontal pathogens profile does not differ significantly in CKD patients compared to non-CKD patients as reported by other studies.

## Figures and Tables

**Table 1:** Clinical characteristics of the study group.

Demographic and clinical parameters	Total (*n* = 70)
Age (years)^*^	46.3 ± 16.1 (18–80)
Sex (% male)	37 (52.9%)
Creatinine (mg/dL)^*^	2.6 ± 1.2 (1.20–8.35)
eGFR^*^ (mL/min/1.73 m^2^)	28.6 ± 10.8 (8–50)
Hb^*^ (g/dL)	10.9 ± 1.95 (7.10–16.90)
Serum albumin (g/dL)^*^	3.6 ± 0.5 (1.70–4.60)
Smokers	15 (21.4%)
Diabetes	7 (10%)

eGFR: estimated glomerular filtration rate; Hb: hemoglobin; ^*^values as mean ± standard deviation.

**Table 2:** Periodontal characteristics of the study group.

Periodontal parameters	Total (*n* = 70)
Mean CAL (mm)^*^	3.6 ± 1.1 (1.8–6.1)
Mean PPD (mm)^*^	2.6 ± 0.4 (1.8–3.8)
Sites with BoP (%)^*^	24.8 ± 14.5 (1–82)
PI (%)^*^	90.6 ± 9 (73–100)

CAL: clinical attachment level; PPD: periodontal pocket depth; BoP: bleeding on probing; PI: plaque index; ^*^values as mean ± standard deviation.

**Table 3:** Clinical characteristics of the study group according to presence or absence of periodontal disease.

Parameter	With PD (*n* = 46)	Without PD (*n* = 24)	*P* ^*^
Age (years)	49.2 ± 16.6	40.8 ± 13.8	0.02
Sex (% males)	48%	29%	0.13
BMI	23.6 ± 3.4	22.1 ± 6.4	0.3
Creatinine (mg/dL)	2.7 ± 1.1	2.5 ± 1.6	0.5
eGFR (mL/min/1.73 m^2^)	27 ± 10.4	31.8 ± 11	0.04
Hb (g/dL)	10.7 ± 2.1	11.2 ± 1.3	0.2
Serum albumin (g/dL)	3.7 ± 0.6	3.6 ± 0.6	0.5

^*^Patients with PD versus without PD.

eGFR: estimated glomerular filtration rate; Hb: haemoglobin; PD: periodontal disease.

**Table 4:** Frequency of detection and univariate analyses for the association with periodontal disease.

Periodontal pathogen	Frequency of detection (*n* = 70)	*P* ^*^
*A. actinomycetemcomitans *(%)	5.7	0.1
Red complex		
* P. gingivalis* (%)	72.9	0.2
* T. denticola* (%)	85.7	0.001
* T. forsythia* (%)	77.1	0.000
Orange complex		
* P. intermedia* (%)	80	1.0
* P. micros* (%)	91.4	0.003
* F. nucleatum* (%)	88.6	0.2
Yellow complex		
* E. nodatum* (%)	32.9	0.8
Green complex		
* C. gingivalis* (%)	97.1	0.9

^*^ *P* value for the association with PD.

**Table 5:** Distribution of periodontal pathogens according to a 3-range quantification.

Periodontal pathogen	Number of bacteria		
	0	≤10^5^	>10^5^
*A. actinomycetemcomitans* (%)	94.3	5.7	0

*P. gingivalis* (%)	27.1	31.7	41.2
*T. denticola* (%)	14.3	55.9	29.8
*T. forsythia* (%)	22.9	61.8	15.3

*P. intermedia* (%)	20	55.9	24.1
*P. micros* (%)	8.6	91.4	0
*F. nucleatum* (%)	11.4	76.5	12.1

*E. nodatum* (%)	67.1	32.9	0

*C. gingivalis* (%)	2.9	61.8	35.3

**Table 6:** Multivariate analyses for the independent risk factors for PD.

Parameter	*P*	Exp(*B*)	95% C. I. for EXP(*B*)	
			Lower	Upper
*T. denticola *	0.9	0.084	0.001	0.289
*T. forsythia *	0.008	0.014	0.001	0.332
*P. micros *	0.1	0.104	0.004	2.73
Age (years)	0.04	1.086	1.003	1.175
eGFR (mL/min/1.73 m^2^)	0.9	1.004	0.903	1.117

eGFR: estimated glomerular filtration rate.
